# The effect of clinical-grade retinal pigment epithelium derived from human embryonic stem cells using different transplantation strategies

**DOI:** 10.1007/s13238-018-0606-8

**Published:** 2019-01-23

**Authors:** Lei Wang, Wei Wu, Qi Gu, Zengping Liu, Qiyou Li, Zhongwen Li, Jinhui Fang, Wenjing Liu, Jun Wu, Ying Zhang, Liu Wang, Haiwei Xu, Wei Li, Baoyang Hu, Qi Zhou, Zhengqin Yin, Jie Hao

**Affiliations:** 1 Savaid Medical School, University of Chinese Academy of Sciences, Beijing, 100049 China; 2 State Key Laboratory of Stem Cell and Reproductive Biology, Institute of Zoology, Chinese Academy of Sciences, Beijing, 100101 China; 3 Southwest Hospital/Southwest Eye Hospital, Third Military Medical University (Army Medical University), Chongqing, 400038 China; 4 Beijing Stem Cell Bank, Chinese Academy of Sciences, Beijing, 100190 China; 5 State Key Laboratory of Membrane Biology, Institute of Zoology, Chinese Academy of Sciences, Beijing, 100101 China; 6 Stem Cell and Regenerative Medicine Innovation Institute, Chinese Academy of Sciences, Beijing, 100101 China

**Dear Editor,**

Many forms of sight-threatening diseases, including retinitis pigmentosa (RP) and age-related macular degeneration (AMD), are caused by the dysfunction, degeneration and loss of the retinal pigment epithelium (RPE) (Strauss, 2005). RPE cell transplantation may potentially recover or halt disease progression, in which human embryonic stem cells (hESCs) could serve as an unlimited donor source for RPE differentiation, and a few clinical trials have shown the safety and effective of transplantation of hESCs-derived RPE (hESC-RPE) for AMD patients (Schwartz et al., 2012; Schwartz et al., 2015; Song et al., 2015; da Cruz et al., 2018; Kashani et al., 2018; Liu et al., 2018).

However, a report about vision loss after intravitreal injection of autologous stem cells for AMD implied strategic and technical problems remaining with RPE cell therapy (Kuriyan et al., 2017). The quality control of donor cells is basical requirement for cell production in clinical trials. Firstly, the cell treatment products should not be exposed to animal products to minimize the risk of animal pathogen-derived infection and immunologic rejection. Besides, stem cell residues and chromosome number variation during long-term culture must be tested before clinical use.

In addition to quality control of donor cells, the transplantation methodology is also very important. So far, hESC-RPE cells were delivered into the subretinal space by means of RPE cells suspensions and cell sheet. Although both the two strategies have been proven feasible, safe and effective in the previous clinical trials (Schwartz et al., 2012; Schwartz et al., 2015; Song et al., 2015; da Cruz et al., 2018; Kashani et al., 2018; Liu et al., 2018), the comparison between these two strategies should be well studied to maximize the effects of RPE transplantation.

Previously, we have established a clinical-grade hESC line (Q-CTS-hESC-2) (Gu et al., 2017) RPE (Q-CTS-hESC-2-RPE) cells from which have been demonstrated safety and feasibility for wet-AMD (Liu et al., 2018). In the present study, we standardized the preparation of Q-CTS-hESC-2-RPE cells under conditions compliant with good manufacturing practice (GMP) and identified the characterization of Q-CTS-hESC-2-RPE cells in terms of biosafety, genetic safety and cellular function. Furthermore, we compared the RPE cells suspensions and cell sheet transplantation using a well-known model of dry AMD, Royal College of Surgeons (RCS) rats. Our study might facilitate the clinical translation of RPE cells suspensions and cell sheet transplantation for retinal degeneration diseases.

As shown in Fig. 1A, we differentiated the Q-CTS-hESC-2 cells into RPE cells using spontaneous differentiation protocol (Maruotti et al., 2013). Adherent Q-CTS-hESC-2 colonies without feeder cells were continuously cultured to generate pigmented cells (Fig. S1A and S1B), which were subsequently enriched and passaged until the formation of typical cobblestone-like RPE cells (Fig. S1C). During the differentiation process, we collected cells at different stages for the expression comparison of related genes. Results of reverse-transcription quantitative polymerase chain reaction (RT-qPCR) showed a downregulation of *OCT4* (pluripotency marker) and significant expression of *OTX2*, *MITF* and *RPE65* (RPE markers) in the Q-CTS-hESC-2-RPE cells compared to the cells in the stem-cell state and differentiated state (hESCs on 45 days post differentiation were named as ES-45) (Fig. S1D). Consistently, flow cytometry of Q-CTS-hESC-2-RPE cells revealed scarce expression of OCT4 and ubiquitous expression of BEST1, MITF and RPE65 (Fig. 1B–E). Transmission electron microscopy demonstrated the typical ultrastructure of RPE cells, including apical villi (AV), tight junctions (TJ) and melanin granules (MG) (Fig. 1F). After a long-time culture, Q-CTS-hESC-2-RPE cells remained a normal female karyotype (46, XX) (Fig. 1G). Also, copy number variation (CNV) sequencing indicated that no chromosome aneuploidy and no DNA loss or repeat greater than 10 Mbps in the Q-CTS-hESC-2-RPE cells (Fig. 1H).

**Figure 1:**
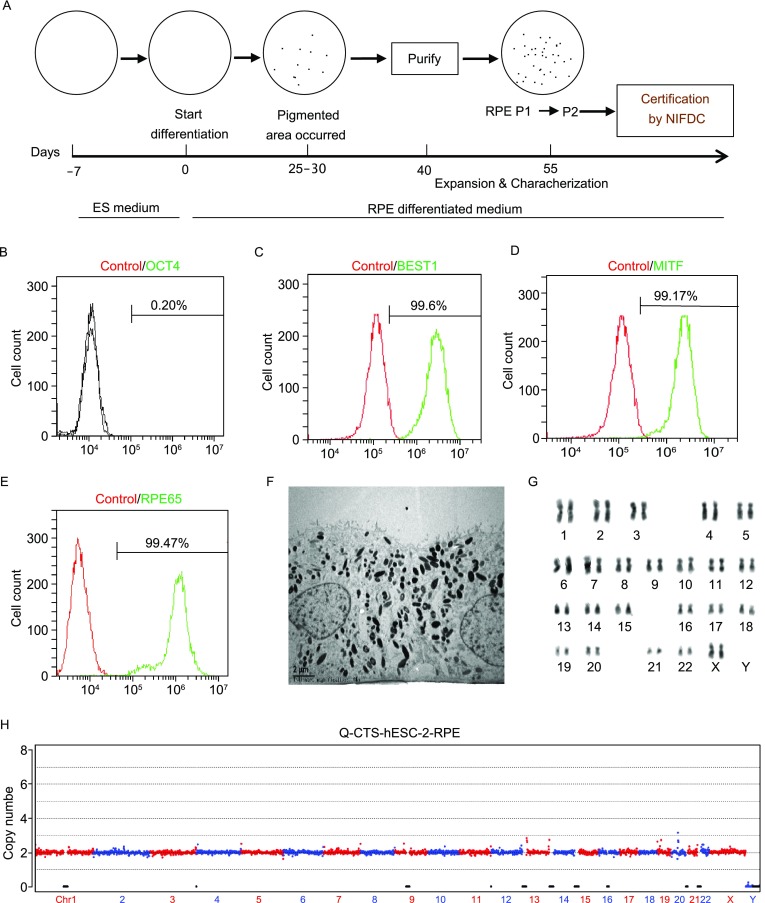
**Characterization of Q-CTS-hESC-2-RPE cells**. (A) Timecourse of the differentiation of Q-CTS-hESC-2-RPE cells. Flow cytometric analysis the expression of OTC4 (B) and key RPE markers BEST1 (C), MITF (D) and RPE65 (E). (F) Ultrastructure of the Q-CTS-hESC-2-RPE cells. Scale bar = 2 μm. (G) Karyotype analysis of Q-CTS-hESC-2-RPE cells. (H) Whole-genome CNV detection of Q-CTS-hESC-2-RPE cells. Abbreviations: NIFDC, National Institutes for Food and Drug Control of China

To evaluate the biosafety of Q-CTS-hESC-2-RPE cells, we firstly performed the teratoma formation assays, which indicated no teratomas generation after injecting Q-CTS-hESC-2-RPE cells into the severe combined immunodeficiency (SCID) mice (Fig. S1E, S1F and Table S1). Furthermore, we performed a serious test according to the Guidance of Human Somatic Cell Therapies and Quality Control of Cell-based Products. The results demonstrated that the Q-CTS-hESC-2-RPE cells were negative for mycoplasma and free of serious pathogenic microorganisms (Table S2), which met the requirements of Pharmacopoeia of the People’s Republic of China, 2010 edition, Volume III. These results indicated that the Q-CTS-hESC-2-RPE cells were biologically safe. Importantly, Q-CTS-hESC-2-RPE cells also met the present clinical cell application standard in China, and we obtained a qualification (report number SH201502158) from the National Institutes for Food and Drug Control of China (Table S3).

For cell suspension transplantation, the Q-CTS-hESC-2-RPE cells were expanded on a regular culture plate. To achieve cell sheet transplantation, the donor cells were cultured on polyethylene terephthalate (PET) membrane, which has been used as a carrier to deliver monolayers of RPE cells (Stanzel et al., 2014; da Cruz et al., 2018). Immunostaining revealed the Q-CTS-hESC-2-RPE cells expressing RPE markers PAX6, ZO-1 and BEST1 on a culture plate and PET membrane (Fig. 2A–C and 2A′–C′). To detect the capacity to phagocytize POS, we cultured Q-CTS-hESC-2-RPE cells with neural retinas of rats for 48 h. The immunostaining of RHODOPSIN and F-ACTIN which labeled POS and cytoskeleton respectively and the orthogonal views of stacking images indicated that POS was internalized by the Q-CTS-hESC-2-RPE cells on culture plate and PET membrane (Fig. 2D and 2D′). Finally, the enzyme linked immunosorbent assay (ELISA) confirmed a robust secretion of pigment epithelium-derived factor (PEDF) in the Q-CTS-hESC-2-RPE cells on culture plate and PET membrane, while undifferentiated hESCs could not secrete PEDF. Notably, Q-CTS-hESC-2-RPE cells on PET membrane secreted more PEDF than that on culture plate (Fig. S1G).

**Figure 2:**
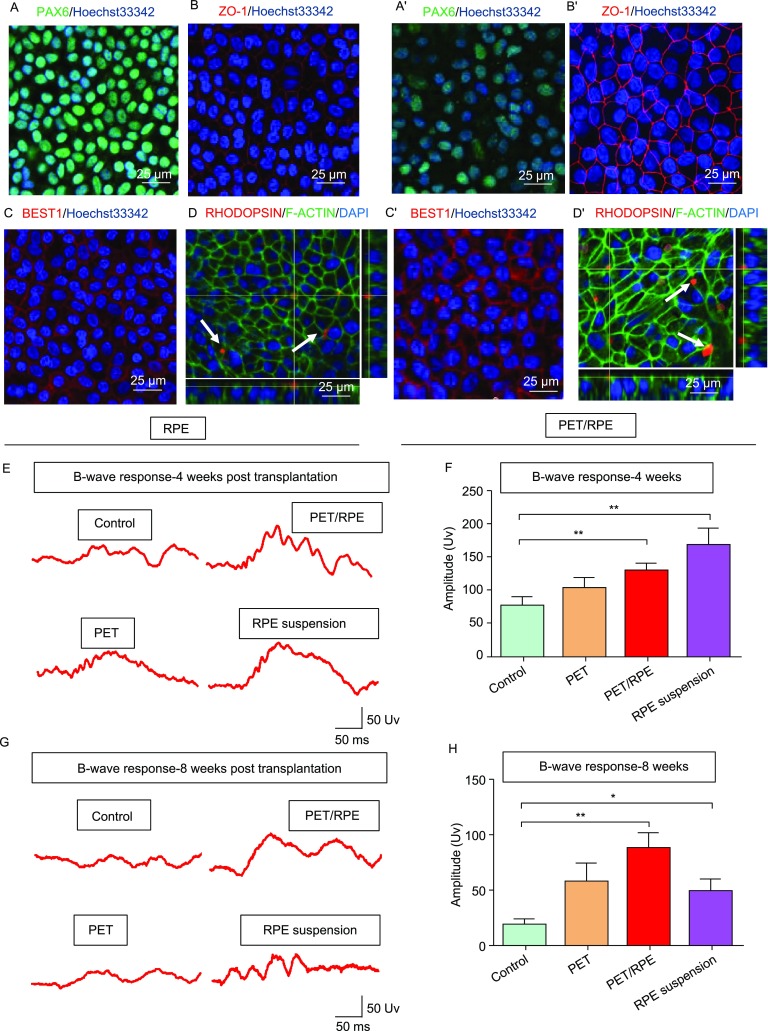
**Comparison of the Q-CTS-hESC-2-RPE cells on a culture plate and PET membrane** ***in vitro*** **and** ***in vivo***. (A–C) Immunostaining of the typical RPE markers of PAX6 (A), ZO-1 (B) and BEST1 (C) in Q-CTS-hESC-2-RPE cells on culture plate. Nuclei were counterstained with Hoechst 33342 (blue). Scale bars = 25 μm. (D) Orthogonal views of fluorescence images showed the location of internalized POS in Q-CTS-hESC-2-RPE cells on culture plate. RHODOPSIN (red) was used to show the POS, while F-ACTIN (green) was used to show the cell morphology. Nuclei were stained with DAPI (blue). Arrows indicated the POS. Scale bars = 25 μm. (A’–D’) Same as (A–D), but for the Q-CTS-hESC-2-RPE cells on PET membrane. (E) The representative ERG traces in the control, PET, PET/RPE and RPE suspension groups at 4 weeks after transplantation. (F) Data statistics of amplitude of ERG B wave in the control (*n* = 11), PET (*n* = 11), PET/RPE (*n* = 14) and RPE suspension (*n* = 11) groups at 4 weeks after transplantation. (G–H) Same as (E–G), but for 8 weeks after transplantation

We next delivered the Q-CTS-hESC-2-RPE cells into the subretinal space of RCS rats using two approaches: cell suspension and cell sheet transplantation, while acellular PET membrane transplantation and untreated RCS rats served as controls. Intravital examination of fundus photograph confirmed a successful transplantation of PET/RPE patch, acellular PET membrane and Q-CTS-hESC-2-RPE cells suspension (Fig. S2A, S2C and S2E). Consistently, hematoxylin-eosin (HE) staining showed that the PET/RPE patch, acellular PET membrane and clumped Q-CTS-hESC-2-RPE cells suspension were located in the subretinal space of RCS rats (Fig. S2B, S2D and S2F). Notably, there were clear distortions of retinal lamination after the transplantation of PET/RPE and acellular PET patch as the rigidity of PET membrane mismatch with the soft and curvate retina. Finally, electroretinogram (ERG) assay was used to evaluate the function of the retina on 4 and 8 weeks post transplantation. For both time points, the amplitude of the ERG B wave in PET/RPE group and the cells suspension group were significantly higher than that in the acellular PET and untreated group (Fig. 2E–H), which suggested that both Q-CTS-hESC-2-RPE cell sheet and cell suspension transplantation significantly rescued retinal degeneration. However, there is no significant difference between the PET/RPE group and the RPE cells suspension group.

The present study demonstrates the production and transplantation of functional RPE cells for clinical application. To avoid potential infection or contamination, clinical-grade donor cells and reagents are required. Besides, the preparation and treatment of donor cells should be under GMP environments (Andrews et al., 2014). However, in the previous clinical trials with small samples (Schwartz et al., 2012; Schwartz et al., 2015; Song et al., 2015), the donor cells are not strictly clinical grade though the results of the clinical trials have shown safety of hESC-RPE cells transplantation. The safety concerns of donor cells deserve more attention in the following large-scale clinical trials. In this study, we used the clinical-grade hESC line (Q-CTS-hESC-2) to induce RPE cells by well-established spontaneous differentiation, which requires minimal additives to diminish the risk of contamination, infection and pathogen transmission. Notably, all components of the culture and cryopreservation medium, and all processes involved, have been described and validated according to the GMP quality system (Unger et al., 2008). The CTS (Cell Therapy Systems)-grade reagents used in this paper were manufactured by state-of-the-art cGMP- and IOS-certified facilities to ensure the highest quality and consistency for reproducible results. As expected, the Q-CTS-hESC-2-RPE cells have passed a series of strict biosafety tests. Notably, the Q-CTS-hESC-2-RPE cells also showed capacity to form tight junction, phagocytize POS and secrete trophic factors *in vitro*.

RPE suspension transplantation is widely embraced due to the easy operation, but is disadvantaged by poor cell survival and widespread apoptosis. Although RPE cell sheet transplantation is more complicated and limited by the scaffold and surgical tool, the delivery of RPE patch allows the anatomic integration and reliable cell survival. However, only few reports have compared the two transplantation strategies and shown that hESC-RPE cultured on a synthetic parylene substrate survived longer compared to suspension transplantation according to the histological test results (Diniz et al., 2013). Our study compared the two transplantation strategies in terms of visual function which demonstrated is no significant difference of the two groups according to the ERG results. However, the quantity of hESC-RPE cells for suspension transplantation (1 × 10^5^ cells) was about 25 times as compared to cell sheet transplantation (~4000 cells). Due to the small size of RCS rats’ eyes, the cell patch was limited to 1 mm × 0.5 mm with ~4000 cells. For clinical practice, a cell patch of ~ 3 mm × 6 mm with ~1 × 10^5^ cells is used (da Cruz et al., 2018). As a result, it is plausible to speculate that cell sheet transplantation will be more effective than cell suspension transplantation under the same condition. In the future, more comparisons between the two transplantation strategies in terms of anatomic integration and survival rate of grafted cells are required.

## Electronic supplementary material

Below is the link to the electronic supplementary material.

Supplementary material 1 (PDF 421 kb)
