# Supplementary material for: The effect of clinical-grade retinal pigment epithelium derived from human embryonic stem cells using different transplantation strategies
Source: Protein Cell. 2019 Jan 23;10(6):455–60. doi: 10.1007/s13238-018-0606-8 (PMC6538583; doi:10.1007/s13238-018-0606-8)
Supplement: Supplementary file 1 — Supplementary material 1 (PDF 421 kb) [file 13238_2018_606_MOESM1_ESM.pdf]

Supplementary materials

Table S1 Tumorigenic analysis of Q-CTS-hESC-2-RPE

Cell line	Passage number	Cell number	Number of receptors	Number of tumor formation
Q-CTS-hESC-2	P36	1×10^6	5	5/5
Q-CTS-hESC-2-RPE	P2	1×10^6	5	0/5

Table S2 Sterility and pathogen testing for Q-CTS-hESC-2-RPE cells

Sterility and pathogen	Results
Mycoplasma	-
Human papillomavirus (HPV)	-
Human parvovirus B19	-
Human immuno deficiency virus I (HIV- I)	-
Human immuno deficiency virus II (HIV- II)	-
Epstein-barr virus (EBV)	-
Human hepatitis C virus (HCV)	-
Human hepatitus A virus (HAV)	-
Human cytomegalo virus (HCMV)	-
Human hepatitis B virus (HBV)	-
Human herpes virus 6 (HHV-6)	-
Human herpes virus 7 (HHV-7)	-
Bovine parvovirus	-
Porcine parvovirus	-
Swine influenza virus H1N1	-
Bovine serum albumin residuals	< 5 ng/ml
Telomerase	-
Transcriptase	-
Endotoxin level	< 0.5 EU/ml

Table S3 Biological analysis of Q-CTS-hESC-2-RPE cells$

Sterility and pathogen	Q-CTS-hESC-2-RPE
Short tandem repeats (STRs)	Each STR locus has 1-2 alleles
Isozyme analysis	B-type of human origin
Species identification and cell cross-contamination between species	-
Bacteria and fungi	-
Mycoplasma	-
Human papillomavirus (molecular hybridization)	-
Red blood cells adsorption experiments	Negative
Red blood cell agglutinate	Negative
Exogenous virus check-in vitro	Negative
Hemagglutination test of 9- to 11-day-old chick embryo allantoic fluid	Negative
Survival rate of 5- to 6-day-old chick embryos	100%
Intracerebral and intraperitoneal injections in suckling mice	Survival rate 80%
Intracerebral and intraperitoneal injections in mice	Survival rate 100%
Intraperitoneal injection in guinea pigs	Survival rate > 90%
Intracutaneous and subcutaneous injections in rabbits	Survival rate > 90%
Immunological reaction test	RPE:PHMC = 1:5, Inhibition rate = 98%
Expression of OTX2, BEST1, ZO-1, MITF, RPE65	Positive
Secretion of PEDF	3.1 ng/Ml
Melanin granules in the cytoplasm	Yes
Positive cells proportion of TRA-1-60, TRA-1-81, OCT4 by FACS	< 1%
Expression of OCT4, NANOG by Immunofluorescence staining	Negative
Expression of OCT4, NANOG by real-time PCR	Negative
Tumorigenicity	Negative
The cell survival rate	> 90%
Sub-G1 (%)	0
GO/G1 (%)	76.54
S (%)	12.34
G2/M (%)	10.05

Soft AGAR clone formation test	No clone formation
Telomerase activity (TPG/10000 cells)	< 2
Bovine serum albumin residuals	< 5 ng/ml
Endotoxin level	< 0.5 EU/ml

$ This table is translated from NIFDC report numbers, SH201502158 for Q-CTS-hESC-2-RPE.

Table S4 Primer sequences

Gene	Primer sequence (5' to 3')	Annealing Temp
OCT4	F- GTGGAGGAAGCTGACAACAA	60℃
	R- ATTCTCCAGGTTGCCTCTCA	
OTX2	F- ACCTTGAACTCCACCTCT	60℃
	R- GCTTCTCTTCTCTGACTCTCTTTG	
MITF	F- TTCACGAGCGTCCTGTATGCAGAT	60℃
	R- TTGCAAAGCAGGATCCATCAAGCC	
RPE65	F- AAATGTCTATCCAGGTTGAGCA	60℃
	R- GCTTGCCCATCAAACAGGTG	

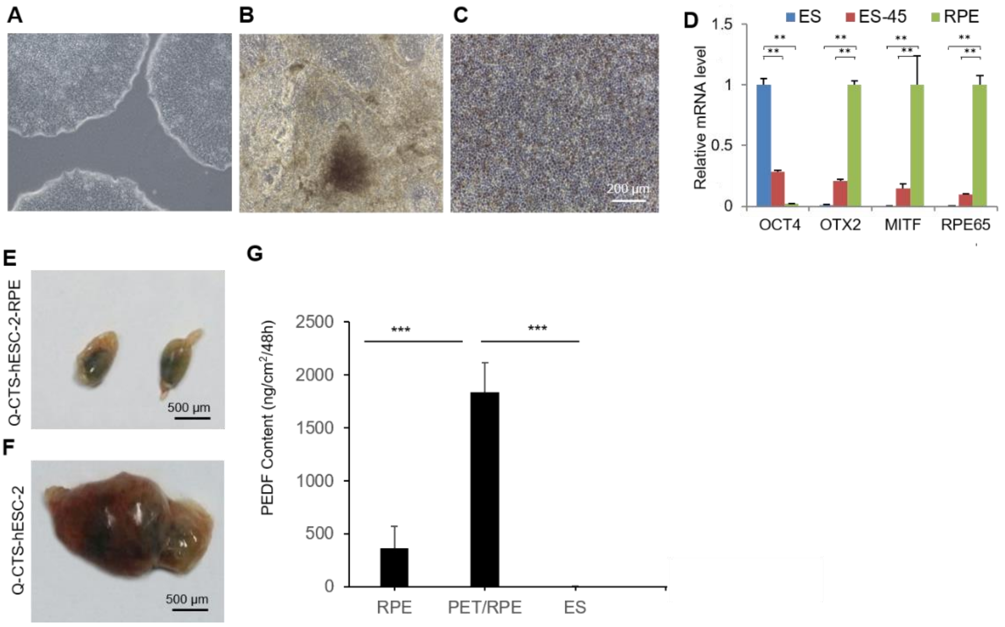

Supplementary Figure 1. Characterization of differentiated hESC-RPE cells.

(**A-C**) Phase contrast images of the cells during differentiation with stages for hESC (**A**), pigmented cells (**B**), and hESC-RPE (**C**), scale bars, 200 μm.

(**D**) Comparative gene expression of *OCT4, OTX2, MITF* and *RPE65* among differentiated RPE (RPE) stage, hESCs stage , and differentiation stage of cells on day 45 post differentiation (ES-45). Relative gene expression represents data normalized to GADPH and expressed relative to RPE. Error bars represent ± SEM, *P < 0.05, **P < 0.01, n = 3.

(E, F) Teratoma test for the differentiated hESCs cells (E) compared to RPE (F). Scale bars = 500 μm.

(G) Enzyme-linked immunosorbent assay analysis of PEDF. The secretion of PEDF on PET membrane and on culture plate. The PEDF level of PET/PRE was significantly higher than that of ES (negative control) and RPE on the plate. Mean ± S.D, ***P < 0.01, n = 3.

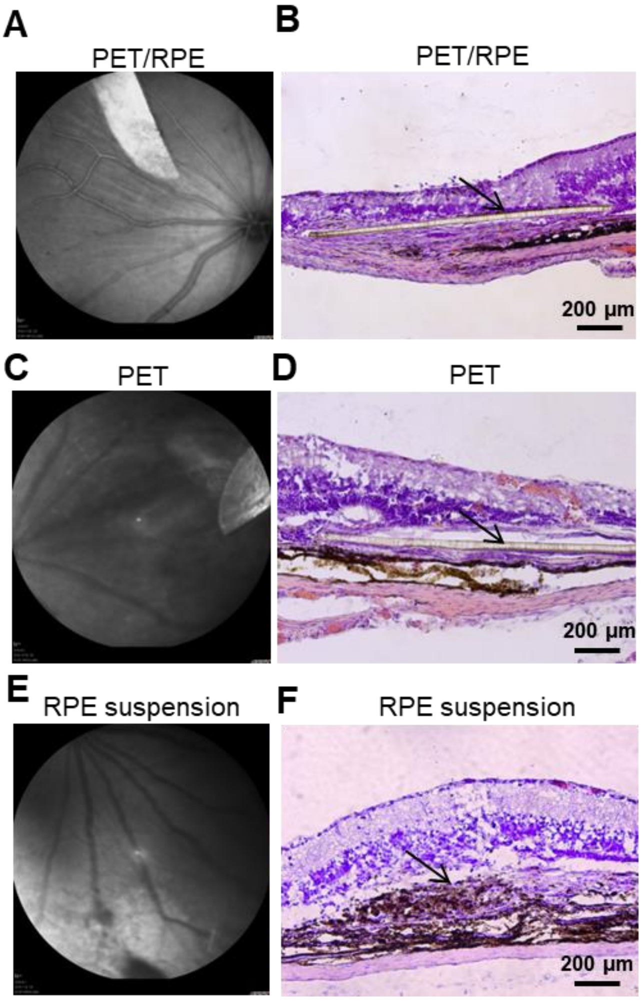

Supplementary Figure 2: The morphological detection of the retinas of RCS rats post transplantation.

Fundus photograph and HE staining showed the transplanted areas of PET/RPE (A, B), PET membrane (C, D) and RPE suspension (E, F) group at 2 weeks after transplantation.

Materials and methods

Human embryonic stem cell culture

The human embryonic stem cell line we used in this research (Q-CTS-hESC-2) was derived using GMP conditions in our lab (Gu et al., 2017) and were maintained on rhVTN-N (Thermo Fisher, USA, A14700)-coated plates in E8 medium (Thermo Fisher, USA, A1517001), which is a chemically defined medium for hESC culture (Chen et al., 2011), in a 5% CO_2 and 20% O_2 incubator.

Differentiation and culture of clinical-grade RPE from hESCs

RPE differentiation method was referenced to the previous research (Maruotti et al., 2013). The hESC colonies were expanded for 7-10 days in E8 medium. When observing colony attached to each other, changed E8 medium to RPE differentiation medium (RPE-DM) consisting of CTS™ KnockOut Dulbecco's Modified Eagle's Medium (CTS-KO-DMEM, Thermo Fisher, USA, A1286101), 20% CTS™ KnockOut SR Xeno-Free Medium (CTS-KOSR, Thermo Fisher, USA, 12618013), 2 mM Glutamax CTS™ 100X (Thermo Fisher, USA, A1286001), 1 mM nonessential amino acids (NEAA, Thermo Fisher, 11140050). The medium was refreshed every day during the first week followed by refreshing every other day. Approximately 25-30 days later, pigmented area became visible, and pigmented areas enlarged after an additional 30 days' culture. If the pigmented areas were manually collected and plated on rhVTN-N-coated culture plates in RPE-DM, and the medium was changed every other day. Passaging was performed every two weeks.

PCR analysis

Total RNA extraction and reverse transcription were performed as previous research (Wang et al., 2013). Real-time PCRs were conducted with the SYBR® Premix EX Taq™ kit (Takara, Japan) and a Mx3005P (Agilent, Santa Clara, California, USA) according to the manufacturer's instructions. Primer sequences used in the experiments are presented in Supplementary Table 4. GAPDH was an internal reference for normalization.

Karyotyping and G-banding

When clinical-grade hESC-RPE cells reached 80-90% confluency, we sent the cells to Chinese Academy of Medical Science and Peking Union Medical College. G-banding analysis was performed by the Chinese Academy of Medical Science and Peking Union Medical College.

Copy number variation (CNV) sequencing

When the RPE cells were cultured for four generations, the cells were harvested by digestion using CTS™ TrypLE™ Select Enzyme (Thermo Fisher, USA, A1285901). Next, approximately 5 x 10^6 cells were collected and delivered to YIKON GENOMICS company (Tai Zhou, Jang Su, China) to perform a CNV test.

Flow cytometry

hESC-RPE cells with P3 were harvested by treatment with CTS™ TrypLE™ Select Enzyme,

and the cells were suspended in DPBS (Thermo Fisher, USA). Samples were incubated with primary antibodies OCT4 (mouse, 1/200 Santa Cruz, USA), MITF (goat, 1/200, Santa Cruz, USA), BEST1 (rabbit, 1/200, Abcam, USA), and RPE65 (mouse, 1/200 Abcam, USA). After rinsing, the samples were incubated with secondary antibodies donkey anti-mouse IgG (1/200, Jackson, USA), donkey anti-goat IgG (1/200, Jackson, USA), and donkey anti-rabbit IgG (1/200, Jackson, USA). The cells in the control group were only incubated with the secondary antibody. After washing three times, the cells were analysed on a flow cytometer (Beckman Coulter, cytoFLEX).

Teratoma formation

When the hESCs reached 80% confluence and the RPE cells were purified for two generations, the cells were digested using CTS™ TrypLE™ Select Enzyme (Thermo Fisher, USA, A1285901) and suspended in CTS-DPBS (Thermo Fisher, USA, A12858-01) at a density of 5 x 10^7 cells/mL. Following collection, 20 μL of cell suspension was carefully injected into each testis of 6- to 8-week-old SCID mice using a sterile glass needle under a sterile stereo microscope. 6 weeks later, according to the guidelines of the Institutional Animal Care and Use Committee, the mice were euthanized, and the teratomas were examined. The teratomas were then fixed and stained with H&E for histology analysis.

Transmission electron microscopy

This method was performed according to our previous protocol (Wei Wu, 2016). Briefly, hESC-RPE cells on Transwell filters were fixed them with 2.5% glutaraldehyde in 0.1 M cacodylate buffer (pH 7.4) overnight. Then, the samples were washed twice with 0.1 M PBS followed by being post-fixed with 1% osmium tetroxide for 2 hours and washed twice with 0.1 M PBS. We used increasing concentrations of acetone to dehydrate samples and embedded the samples in epoxy resin 618. Finally, a diamond knife (Diatome, USA) was employed to make ultrathin sections, and then the sections were stained with 2% lead citrate and 2% uranyl acetate before examination in a JEM-1400 Plus (JEOL) electron microscope.

Immunostaining

After fixation by 4% paraformaldehyde (PFA), the cells were blocked and permeabilized for 10-20 min in 2% bovine serum albumin (BSA, Sigma-Aldrich, USA), 0.25% Triton X-100 (Solarbio, China) in PBS and then incubated overnight at 4 ℃ with the following primary

antibodies: rabbit anti-OTX2 1/200 (Millipore, USA), rabbit anti-ZO-1 1/200 (Thermo Fisher, USA), rabbit anti-BEST1 1/200 (Abcam, USA), and then the cells were incubated for 1 hour with the corresponding secondary antibody conjugated to Alexa 488 or CY3 1/200 (Jackson, USA) and counterstained with Hoechst 33342 (Thermo Fisher, USA) for 15 min. All labelled cells were imaged with a laser-scanning confocal microscope (Carl Zeiss, LSM710).

Phagocytosis assay

The phagocytosis assay was performed according to our previous protocol (Wu et al., 2016). In brief, the neural retinas of non-dystrophic RCS rats at age 8 weeks were peeled and co-cultured with RPE cells or PET/RPE for 72 h. Then the retinal explants were removed, and RPE cells and PET/RPE were analyzed by immunostaining according to the protocols above. The primary antibody of anti-Rhodopsin (ab81702, 1:200, Abcam) was used to detect the internalized photoreceptor outer segments (POS), while FITC-conjugated phalloidin (P5282, Sigma-Aldrich) was used to show the cell morphology. Finally, the RPE cells and PET/RPE were imaged with a laser-scanning confocal microscope (Carl Zeiss, Germany, LSM 710).

Enzyme-linked immunosorbent assay (ELISA) for pigment epithelium-derived factor (PEDF)

Clinical-grade hESC-RPE cells and hESCs were cultured for 72 hours respectively. Then, the cell culture medium and fresh RPE medium were collected, and the level of PEDF level was detected using a PEDF ELISA kit (Millipore, USA) according to the manufacturer's instructions. All conditioned medium samples were run in technical duplicate.

Biological safety detections

Biological safety detections were performed as described previously for our clinical-grade human induced pluripotent stem cells, including endotoxin testing, mycoplasma testing, and pathogenic microorganism testing (Wang et al., 2015).

Transplantation of hESC-RPE cells into RCS rats

For cell sheet transplantation, the hESC-RPE cells were cultured on PET membranes (Transwell, Corning) for 2 weeks. Before transplantation, PET/RPE and acellular PET implants were trephined into 1 × 0.5 mm^2 sections by a custom trephine. Royal college of surgeons (RCS) rats (P21, regardless of sex) were maintained on a 12 h light/dark cycle. The animals were anaesthetized using a mixture of 10 mg/kg ketamine (Sigma-Aldrich, St. Louis,

MO, USA) and 1 mg/kg xylazine (Sigma-Aldrich), and the pupils were pharmacologically dilated. A peritomy exposed the superior temporal pole of the sclera. Approximately 1.5 mm posterior to the limbus, the sclera and choroid were incised by a 27-gauge needle. A retinal detachment was created by injecting 10 μL of sterile balanced salt solution into the subretinal space with a 33-gauge blunt needle. Then, the PET/RPE implant or PET implant was inserted into the subretinal space with fine forceps. The scleral and conjunctiva aperture were closed by 10-0 sutures. Finally, tobramycin dexamethasone eye ointment was administered to prevent infection.

For cell suspension transplantation, 1×10^5 cells in 1 ~ 2 μL of PBS was injected into the subretinal space through a small scleral incision with a fine glass pipette (Hamilton). The cornea was punctured to reduce intraocular pressure and to reduce the efflux of cells. The rats were immunosuppressed by the addition of 210 mg/L cyclosporine A (Sandoz, Camberley) to the drinking water 24 hours before the transplantation. The rats then remained on the same immunosuppressant throughout the experiment.

HE staining

After fixation with 4% PFA, the tissue was dehydrated with 70%, 80%, 90%, and 100% ethanol sequentially for 1 h in each solution. Next, the samples were submerged by the xylene, next by permeated with paraffin and xylene solution. Then, the samples were infiltrated with the paraffin overnight and embedded by paraffin. Finally, we sectioned the sample for further imaging.

Reference

Chen, G., Gulbranson, D.R., Hou, Z., Bolin, J.M., Ruotti, V., Probasco, M.D., Smuga-Otto, K., Howden, S.E., Diol, N.R., Propson, N.E., *et al.* (2011). Chemically defined conditions for human iPSC derivation and culture. Nat Methods 8, 424-429.

Gu, Q., Wang, J., Wang, L., Liu, Z.X., Zhu, W.W., Tan, Y.Q., Han, W.F., Wu, J., Feng,

C.J., Fang, J.H., *et al.* (2017). Accreditation of Biosafe Clinical-Grade Human Embryonic Stem Cells According to Chinese Regulations. Stem Cell Reports 9, 366-380.

Maruotti, J., Wahlin, K., Gorrell, D., Bhutto, I., Lutty, G., and Zack, D.J. (2013). A simple and scalable process for the differentiation of retinal pigment epithelium from human pluripotent stem cells. Stem Cells Transl Med 2, 341-354.

Wang, J., Gu, Q., Hao, J., Bai, D., Liu, L., Zhao, X., Liu, Z., Wang, L., and Zhou, Q. (2013). Generation of induced pluripotent stem cells with high efficiency from human umbilical cord blood mononuclear cells. Genomics Proteomics Bioinformatics 11, 304-311.

Wang, J., Hao, J., Bai, D., Gu, Q., Han, W., Wang, L., Tan, Y., Li, X., Xue, K., Han, P., *et al.* (2015). Generation of clinical-grade human induced pluripotent stem cells in Xeno-free conditions. Stem Cell Res Ther 6, 223.

Wei Wu, Y.Z., Zhengya Li, Qiyou Li, Haiwei Xu, Zheng Qin Yin (2016). Features specific to retinal pigment epithelium cells derived from three-dimensional human embryonic stem cell cultures - a new donor for cell therapy. Oncotarget 7, 22819-22833.
